# Profiling of psychoneurological symptoms in newly diagnosed head and neck cancer patients

**DOI:** 10.1007/s00520-025-09871-2

**Published:** 2025-08-29

**Authors:** Joan C. Medina, Femke Jansen, Birgit I. Lissenberg-Witte, Remco de Bree, Ruud H. Brakenhoff, Jose Hardillo, Johannes A. Langendijk, C. René Leemans, Robert P. Takes, Femke Lamers, Irma M. Verdonck-de Leeuw

**Affiliations:** 1 Department of Psychology and Education Sciences, Universitat Oberta de Catalunya, Barcelona, Spain; 2 eHealth ICOnnecta’t Program, Institut Català d’Oncologia, L’Hospitalet de Llobregat, Spain; 3 Department of Otolaryngology-Head and Neck Surgery, Amsterdam University Medical Center Location Vrije Universiteit Amsterdam, Amsterdam, The Netherlands; 4 Treatment and Quality of Life Program, Cancer Center Amsterdam, Amsterdam, The Netherlands; 5 Department of Epidemiology and Data Science, Amsterdam University Medical Center Location Vrije Universiteit Amsterdam, Amsterdam, The Netherlands; 6 Department of Head and Neck Surgical Oncology, University Medical Center Utrecht, Utrecht, The Netherlands; 7 Department of Otorhinolaryngology, Erasmus MC Cancer Institute, University Medical Center, Rotterdam, The Netherlands; 8 Department of Radiation Oncology, University Medical Center Groningen, University of Groningen, Groningen, The Netherlands; 9 Department of Otorhinolaryngology-Head and Neck Surgery, Radboud University Medical Center, Nijmegen, The Netherlands; 10 Department of Psychiatry, Amsterdam University Medical Center Location Vrije Universiteit Amsterdam, Amsterdam, The Netherlands; 11 Mental Health Program, Amsterdam Public Health, Amsterdam, The Netherlands; 12 Department of Clinical, Neuro and Developmental Psychology, Vrije Universiteit Amsterdam, Amsterdam, The Netherlands

**Keywords:** Mental health, Anxiety, Depression, Screening, Inflammation, Latent class analysis

## Abstract

**Purpose:**

He ad and neck cancer (HNC) can trigger a significant mental health burden, including psychoneurological symptoms (PNS). Better insight into the profiling of PNS is important for advancing personalized mental health screening and management.

**Methods:**

Data from 538 newly diagnosed adult HNC patients participating in a prospective multicenter cohort study (NET-QUBIC) were used. Questionnaires were used to assess PNS. Sociodemographic, clinical, lifestyle, and biological variables were collected. Latent class analysis was performed to identify differential classes of PNS. Between-class comparisons and multivariable logistic regression analyses were conducted to characterize each profile in relation to sociodemographic, clinical, lifestyle, and biological variables.

**Results:**

Fit indexes supported a three-class solution, with patients distributed in mild (60%), moderate (26%), and severe (14%) PNS classes. Pain and sleep problems were featured in all classes, anxiety and depression in the moderate and severe classes, and fatigue only in the severe class. Patients in the moderate and severe classes were more often women, had oral cavity cancer, showed impaired performance, had a history of anxiety and depression disorders, were daily smokers, had higher CRP, and had a flatter cortisol slope compared to the mild class.

**Conclusion:**

Newly diagnosed HNC patients can be classified according to the severity of PNS. Several sociodemographic, clinical, lifestyle, and biological variables are proposed as drivers for early detection and treatment of mental health burden.

## Introduction

All cancers impact mental health [1]. Specifically for head and neck cancer (HNC), physical changes (e.g., oral dysfunction) can exacerbate such impact producing pain or sleep problems [2]. Additionally, there are social effects that must be considered, involving the impact of cancer on interpersonal relationships, employment, and financial well-being [3]. Recently, biological mechanisms underlying mental health burden have also been suggested [4], such as inflammation and alterations in the hypothalamic–pituitary–adrenal (HPA) axis that may follow the stress triggered by cancer diagnosis [5]. Building upon these biological underpinnings, an immunometabolic depression profile has even been characterized [6]. Consequently, mental health among cancer patients must be contextualized at the intersection of numerous biological, sociodemographic, clinical, and lifestyle factors.

In cancer research, mental health burden has been approached with a focus on psychoneurological symptoms (PNS), commonly defined as anxiety, depression, pain, fatigue, and sleep problems [7, 8]. PNS are highly prevalent [1], and they can have a profound impact on patients’ cognition [9], quality of life [2], oncological treatment adherence [10], and possibly even cancer recurrence [11]. Unfortunately, PNS are often underdiagnosed, leading to patients’ suffering and associated costs for health systems [12]. It is important to tackle these symptoms from the beginning of the oncological journey because studies demonstrate that their presence tends to be high immediately after diagnosis and more common in HNC patients than in other cancers, with a prevalence of 40% [1, 2].

However, there is limited research specifically focused on this population [13, 14]. In 2022, Lin et al.[13] investigated networks of PNS in relation to demographic and clinical characteristics. Depression was the core symptom followed by fatigue. Female gender, stress, and no alcohol use were associated with more symptomatology. The same year, Santoso et al. [14] investigated networks of PNS in relation to biomarkers of stress (diurnal cortisol slope), inflammation (c-reactive protein (CRP), interleukin (IL)−6, IL-10, and tumor necrosis factor alpha (TNF-α)), and two covariates (age and body mass index (BMI)). Poor sleep quality, fatigue, CRP, and IL-6 played an important role in the interconnections between PNS and biomarkers of stress and inflammation.

Based on these findings, more detailed understanding of the prevalence, risk factors, and mechanisms underlying PNS profiles in HNC patients is essential for their early detection and management. To understand the configuration of these profiles, some studies have conducted model-based analyses such as latent class analysis (LCA). However, to our knowledge, none focused on HNC. In 2019, Tometich et al. [8] retrieved a two-class model among breast cancer patients, but later studies tended to reach three-class solutions. This is the case for Lewson et al. [15] with a sample of patients with diverse diagnoses (but not HNC), Han et al. [16] with a sample of colorectal cancer patients, or Harris et al. [17] again with breast cancer women. All studies interpreted classes according to PNS manifestation, with more severity associated to poorer outcomes (e.g., quality of life, daily performance).

The objective of this study is to provide a comprehensive examination of PNS profiles in newly diagnosed HNC patients. To this end, we build upon the results of the previous study by our team, which identified the PNS network [14]. Now we extend this work, testing whether PNS can be organized in classes, and if these can be characterized by biological, sociodemographic, clinical, and lifestyle factors. We aim to formulate potential PNS risk profiles, to date largely unknown in HNC, to improve mental health screening in this population.

## Materials and methods

### Patients

This study used baseline (i.e., before the start of treatment) data from the NET-QUBIC project [18]. Patients were recruited between March 2014 and June 2018 in 5 hospitals across the Netherlands according to the following inclusion criteria: (1) ≥ 18 years, (2) diagnosed with squamous cell carcinoma in the head and neck region at any stage, (3) scheduled for treatment with curative intent, and (4) competence in Dutch. Exclusion criteria were (1) diagnoses of lymphoma, skin malignancies, or thyroid cancer; (2) inability to comprehend the questions; and (3) experience of severe psychiatric comorbidities preventing the complete understanding of study procedures and informed consent.

The protocol of the NET-QUBIC project was approved by the ethics committee of the leading hospital (2013.301(A2018.307)-NL45051.029.13). The biobank protocol was also approved by the institutional committee (2018.406). All patients signed an informed consent. For this study, patients who provided complete PNS data were retained.

### Data collection

The eligibility of patients was evaluated by the medical doctor in charge of their treatment. If eligible and consenting to participate, assessments were conducted by a trained fieldworker. These included objective measures, self-reported and hetero-administered instruments, and biological samples. Patients received self-reported instruments via postal mail. Objective and hetero-administered measures were taken, and blood samples were collected during home visits or at the hospital. Tubes for saliva samples and envelopes were provided to patients to take and return the samples to the hospital via postal mail. All data were coded immediately and introduced in the NET-QUBIC data warehouse.

### Instruments

The following measures were used to assess PNS.

- Anxiety and depression were measured with the Hospital Anxiety and Depression Scale (HADS) [19]. The HADS was validated in Dutch with good psychometric properties (Cronbach’s alpha = 0.71–0.90) [20]. Higher scores indicate more severity, the cut-offs of ≥ 7 (anxiety) and ≥ 5 (depression) were applied [21, 22].
- Pain was measured with the oral pain subscale of the HNC specific module of the Dutch European Organization for the Research and Treatment of Cancer Quality of Life Questionnaire (EORTC QLQ-H&N35) [23]. This subscale has proven to be valid and reliable across different samples (Cronbach’s alpha ≥ 0.78)[24]. Higher scores indicate more pain, a cut-off of ≥ 10 was used [25].
- Fatigue was measured with the general fatigue subscale of the Multidimensional Fatigue Inventory (MFI), which offers validity and high reliability (Cronbach’s alpha = 0.84)[26]. Higher scores indicate more fatigue; the mean for cancer patients in the original article (≥ 16) was used as a cut-off.
- Sleep problems were measured with the global score of the Pittsburgh Sleep Quality Index (PSQI), an instrument that has shown good validity and internal consistency (Cronbach’s alpha = 0.83) [27]. Higher scores indicate more disturbance; the cut-off of ≥ 6 was used as recommended by the original article.

The covariates analyzed to characterize PNS profiles were:

- Sociodemographic and clinical information obtained through an electronic case report form or fieldwork interview, including age, gender, education, living arrangements, tumor site and stage, and HPV status for oropharynx tumors.
- Performance was assessed through the Eastern Cooperative Oncology Group status [28], with scores above 0 indicating performance restriction.
- Comorbidity was assessed with the Adult Comorbidity Evaluation index [29], categorized as yes or no.
- Lifetime history of anxiety and depression was assessed with the Composite International Diagnostic Interview [30], categorized as yes or no.
- Alcohol intake was assessed through a 21-item study-specific questionnaire, categorized as excessive alcohol consumption (yes/no) according to the National Institute for Public Health and the Environment criteria [31].
- Tobacco consumption was assessed with a 13-item study-specific questionnaire, categorized into daily smoking (yes/no).
- Body mass index (BMI) was obtained from measurements of weight and height taken during fieldwork assessments.
- Inflammatory markers were measured through blood samples collected also during fieldwork assessments. These included CRP, IL-6, IL-10, and TNF-α. CRP was assessed immediately after reception in the laboratories of participating hospitals. For IL-6, IL-10, and TNF-α, serum samples were processed and stored in the NET-QUBIC biobank (Amsterdam UMC, location VUmc) at –80 °C. Then, they were analyzed using ELISA-based technology.
- Stress was measured through saliva cortisol immediately after awakening, 30 and 60 min afterwards, and at 22:00 (or bedtime). Samples were mailed to the coordinating center, processed and stored at –20 °C. They were analyzed using an isotope-dilution LC–MS/MS method. Diurnal cortisol slope was calculated by subtracting the level at 22:00 from the level at awakening, divided by the hours elapsed [32]. Higher values represent steeper cortisol declines.

### Statistical analyses

All analyses were performed with Mplus v.8.7 [33] and R software v.4.2.1 [34]. Little’s test was used to check if data were missing completely at random (MCAR), and participants with complete and incomplete PNS data were compared. The number of comparisons was controlled through the false discovery rate (FDR) method [35]. Patients who provided complete PNS data were retained.

LCA was performed in Mplus with maximum likelihood estimation. The unconditional model was conducted with PNS as indicator variables, followed by modal assignment of patients to their most likely class according to latent class posterior distribution. Models were specified with 50 maximum iterations, 500 random sets of starting values, and 50 final stage optimizations. Additionally, they were run several times to ascertain global maxima, and bivariate residuals were inspected to check local independence. One to five classes were tested, and model selection was guided by interpretability and examination of a set of model fit criteria: AIC (Akaike’s information criterion, an estimator of prediction error), BIC (Bayesian information criterion, similar to AIC but it applies greater penalty to additional parameters), sample-adjusted BIC (SABIC, similar to BIC but it adjusts the penalty to sample size), Lo-Mendell-Rubin Adjusted Likelihood Ratio Test (LMRT, when fitting one additional class it compares the model with the prior using the adjusted asymptotic distribution of the likelihood ratio statistics), and Bootstrapped Likelihood Ratio Test (BLRT, similar to LMRT but it constructs the likelihood ratio distribution using bootstrapping). In all cases, lower values indicate better fit. Entropy is also reported as a measure of classification diagnostics, with values ≥ 0.60 indicating good model quality. All procedures followed the best practices for LCA, including the inspection of several fit criteria [36].

Then, the database was exported to R. Between-class comparisons were conducted on the PNS measures, sociodemographic, clinical, lifestyle, and biological covariates through chi-squared tests for categorical and Kruskal–Wallis tests for continuous variables, since the latter were non-normally distributed. In these comparisons, FDR was also controlled, and pairwise deletion was used. Post hoc tests were conducted, and their residuals were examined. The effect sizes reported are Cramer’s *V* for categorical and Epsilon-squared (*ε*^*2*^) for continuous variables [37, 38]. Finally, multivariable logistic regression was performed, with class regressed on covariates showing significant differences. The variance inflation factor discarded multicollinearity. This manuscript adheres to STROBE reporting guidelines [39].

## Results

### Patient population

From the 739 patients included in the NET-QUBIC project, 538 provided complete PNS data and were analyzed. Sample size was appropriate to produce reliable results [36]. Missing data in the database were MCAR (*X*^*2*^(1497) = 1514.53, *p* = 0.370), with a missingness rate of 14.90%. However, patients with complete PNS data were more often living with others (*X*^*2*^(1) = 19.43, *p* < 0.001), had HPV more often within oropharynx cases (*X*^*2*^(1) = 5.42, *p* = 0.047), better performance (*X*^*2*^(1) = 8.53, *p* = 0.013), fewer comorbidities (*X*^*2*^(1) = 10.88, *p* = 0.007); and had lower CRP (*X*^*2*^(1) = 6.12, *p* = 0.042), IL-6 (*X*^*2*^(1) = 10.06, *p* = 0.007), and TNF-α (*X*^*2*^(1) = 10.05, *p* = 0.007), as well as higher IL-10 (*X*^*2*^(1) = 5.73, *p* = 0.045).

### Latent class analysis

The fit indexes guiding the number of classes set for the LCA are shown in Table 1, together with entropy. Both 2-class and 3-class solutions received support and were interpretable, but three classes were selected; since three indexes favored this model, the BLRT showed no more improvement of a 4-class model, and it showed the highest entropy.

**Table 1:** Model selection criteria for the latent class analysis

Num. classes	AIC	BIC	SABIC	LMRT	BLRT	Entropy
1	3283.60	3305.04	3289.16	-	-	-
2	3038.79	3085.96	3051.04	250.17 (*p* < 0.001)	− 1636.80 (*p* < 0.001)	0.70
3	3029.50	3102.39	3048.43	20.74 (*p* = 0.122)	− 1508.40 (*p* < 0.001)	0.79
4	3031.49	3130.11	3057.10	9.75 (*p* = 0.135)	− 1497.75 (*p* = 0.167)	0.77
5	3035.63	3159.98	3067.92	7.66 (*p* = 0.445)	− 1492.74 (*p* = 0.250)	0.76

Abbreviations: *AIC* Akaike information criteria, *BIC* Bayesian information criteria, *SABIC* sample-adjusted Bayesian information criteria, *LMRT* Lo-Mendell-Rubin Adjusted Likelihood Ratio Test, *BLRT* Bootstrapped Likelihood Ratio Test

**Fig. 1:**
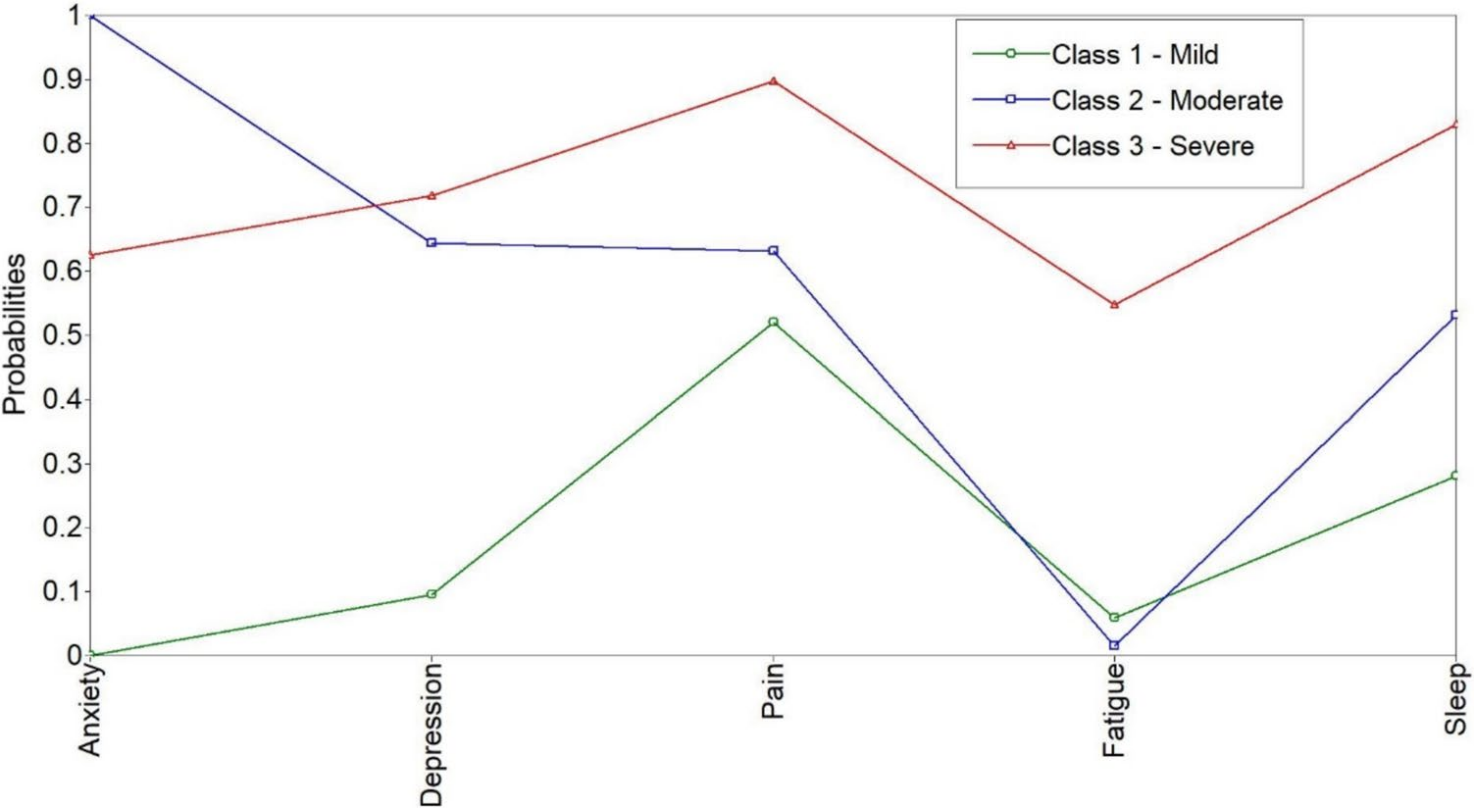
Patterns of psychoneurological symptoms in the three fitted classes

The fitted model showing PNS (*X*-axis) along with item response probabilities (*Y*-axis) is displayed in Fig. 1. Classes can be interpreted as severity levels: mild, moderate, and severe. To some extent, pain and sleep problems are featured in all classes, and depression in the moderate and severe. Fatigue is especially representative of the severe class, while anxiety stands out in the moderate. Most patients (59.67%) were assigned to the mild class, followed by the moderate (25.84%) and severe (14.50%).

### Between-class comparisons

The means and standard deviations (SDs) of participants assigned to each class in all five PNS measures are shown in Table 2. Differences were large for anxiety (*ε*^*2*^ = 0.61), depression (*ε*^*2*^ = 0.40), and fatigue (*ε*^*2*^ = 0.31); and moderate for pain (*ε*^*2*^ = 0.12) and sleep problems (*ε*^*2*^ = 0.23).

**Table 2:** Means and standard deviations for the three classes in all five indicators

	**Mild** (*n* = 321)	**Moderate** (*n* = 139)	**Severe** (*n* = 78)	**KW test**
HADS anxiety	3.14 (1.93)	9.58 (2.46)	7.62 (4.03)	*X*^*2*^(2) = 326.55 (*p* < 0.001)
HADS depression	1.88 (2.16)	5.73 (3.11)	6.59 (3.27)	*X*^*2*^(2) = 216.66 (*p* < 0.001)
EORTC-H&N35 oral pain	20.01 (20.85)	27.52 (22.49)	46.05 (28.45)	*X*^*2*^(2) = 63.96 (*p* < 0.001)
MFI general fatigue	8.63 (4.11)	10.35 (3.26)	16.73 (3.25)	*X*^*2*^(2) = 164.07 (*p* < 0.001)
PSQI total score	4.36 (3.07)	6.78 (3.63)	8.96 (3.32)	*X*^*2*^(2) = 120.88 (*p* < 0.001)

Abbreviations: *KW* Kruskal–Wallis, *HADS* Hospital Anxiety and Depression Scale, *EORTC-H&N35* European Organization for the Research and Treatment of Cancer Quality of Life Questionnaire—Head and Neck Cancer module, *MFI* Multidimensional Fatigue Inventory, *PSQI* Pittsburg Sleep Quality Index

Dunn’s post hoc tests suggested that patients in the mild class, compared to the moderate and severe classes, had lower levels in all PNS (*z* =  − 17.29 – − 3.60, *p* < 0.001). In turn, patients in the moderate class had more anxiety than those in the severe class (*z* = 4.12, *p* < 0.001) but lower pain (*z* =  − 4.37, *p* < 0.001), fatigue (*z* =  − 8.51, *p* < 0.001), and sleep problems (*z* =  − 3.94, *p* < 0.001). There were no differences in depression between these two classes (*z* = 1.21, *p* = 0.228).

The sociodemographic, clinical, lifestyle, and biological variables associated with the three classes are reported in Table 3. Large differences were found for gender (Cramer’s *V* = 0.21), performance (*V* = 0.17), lifetime anxiety (*V* = 0.17), depression (*V* = 0.20), and daily smoking (*V* = 0.18). Moderate differences were found for tumor location (*V* = 0.15). Finally, small differences were found for CRP (*ε*^2^ = 0.02) and Cortisol slope (*ε*^2^ = 0.03).

**Table 3:** Sociodemographic and clinical characteristics of participants in the three classes of the latent class analysis

	**Mild** (*n* = 321)	**Moderate** (*n* = 139)	**Severe** (*n* = 78)	**Chi**^**2**^**/KW test**
Age—mean (SD)	63.99 (9.17)	63.09 (9.74)	61.10 (9.60)	*X*^*2*^(2) = 6.39 (*p* = 0.066)
Women—*n* (%)	59 (18.38%)	44 (31.65%)	33 (42.31%)	*X*^*2*^(2) = 23.05 (*p* < 0.001)
Education level—*n* (%)				*X*^*2*^(4) = 7.50 (*p* = 0.168)
Primary	64 (19.94%)	35 (25.18%)	24 (30.77%)	
Secondary	138 (42.99%)	49 (35.25%)	26 (33.33%)	
Higher	98 (30.53%)	45 (32.37%)	18 (23.08%)	
NA	21 (6.54%)	10 (7.19%)	10 (12.82%)	
Living arrangements—*n* (%)				*X*^*2*^(2) = 1.05 (*p* = 0.617)
Living alone	59 (18.38%)	28 (20.14%)	17 (21.79%)	
Living together	242 (75.39%)	101 (72.66%)	51 (65.38%)	
NA	20 (6.23%)	10 (7.19%)	10 (12.82%)	
Tumor site—*n* (%)				*X*^*2*^(8) = 24.46 (*p* = 0.004)
Oral cavity	73 (22.74%)	47 (33.81%)	28 (35.90%)	
Oropharynx	114 (35.51%)	48 (34.53%)	28 (35.90%)	
Hypopharynx	14 (4.36%)	11 (7.91%)	8 (10.26%)	
Larynx	110 (34.27%)	27 (19.42%)	13 (3.85%)	
Unknown primary	10 (3.12%)	6 (4.32%)	1 (1.28%)	
HPV (oropharynx)—*n* (%)				*X*^*2*^(2) = 1.78 (*p* = 0.468)
Status -	40 (35.09%)	13 (27.08%)	10 (35.71%)	
Status +	60 (52.63%)	30 (62.5%)	12 (42.86%)	
NA	14 (11.97%)	5 (10.42%)	6 (21.43%)	
Tumor stage—*n* (%)				*X*^*2*^(6) = 13.50 (*p* = 0.061)
0–I	87 (27.10%)	33 (23.74%)	11 (14.10%)	
II	60 (18.69%)	17 (12.23%)	18 (23.08%)	
III	42 (13.08%)	26 (18.71%)	18 (23.08%)	
IV	132 (41.12%)	63 (45.32%)	31 (39.74%)	
Performance—*n* (%)				*X*^*2*^(2) = 15.21 (*p* = 0.001)
No restriction	244 (76.01%)	100 (71.94%)	42 (53.85%)	
Some restriction	77 (23.99%)	39 (28.06%)	36 (46.15%)	
Comorbidity—*n* (%)				*X*^*2*^(2) = 3.43 (*p* = 0.216)
No	100 (31.15%)	49 (35.25%)	19 (24.36%)	
Yes	205 (63.86%)	83 (59.71%)	58 (74.36%)	
NA	16 (4.98%)	7 (5.04%)	1 (1.28%)	
Lifetime anxiety—*n* (%)				*X*^*2*^(2) = 12.88 (*p* = 0.004)
No	258 (80.37%)	96 (69.06%)	46 (58.97%)	
Yes	9 (2.80%)	11 (7.91%)	8 (10.26%)	
NA	54 (16.82%)	32 (23.02%)	24 (30.77%)	
Lifetime depression—*n* (%)				*X*^*2*^(2) = 16.41 (*p* < 0.001)
No	240 (74.77%)	82 (58.99%)	40 (51.28%)	
Yes	26 (8.10%)	25 (17.99%)	14 (17.95%)	
NA	55 (17.13%)	32 (23.02%)	24 (30.77%)	
Excess. alcohol con.—*n* (%)				*X*^*2*^(2) = 0.60 (*p* = 0.742)
No	247 (76.95%)	111 (79.86%)	61 (78.21%)	
Yes	73 (22.74%)	27 (19.42%)	17 (21.79%)	
NA	1 (0.003%)	1 (0.01%)	0 (0.00%)	
Daily smoking—*n* (%)				*X*^*2*^(2) = 17.26 (*p* < 0.001)
No	256 (79.75%)	114 (82.01%)	46 (58.97%)	
Yes	64 (19.94%)	24 (17.27%)	31 (39.74%)	
NA	1 (0.003%)	1 (0.01%)	1 (1.28%)	
BMI—mean (SD)	25.91 (4.34)	25.61 (4.86)	25.74 (5.19)	*X*^*2*^(2) = 1.17 (*p* = 0.608)
CRP—mean mg/L (SD)	6.16 (9.92)	5.65 (8.65)	9.69 (16.27)	*X*^*2*^(2) = 8.75 (*p* = 0.023)
IL-6—mean pg/mL (SD)	1.44 (1.29)	1.52 (2.37)	1.78 (1.71)	*X*^*2*^(2) = 3.77 (*p* = 0.202)
IL-10—mean pg/mL (SD)	0.46 (2.00)	1.17 (9.39)	0.38 (0.37)	*X*^*2*^(2) = 3.43 (*p* = 0.216)
TNFα—mean pg/mL (SD)	2.98 (1.66)	2.72 (0.76)	3.57 (3.80)	*X*^*2*^(2) = 4.00 (*p* = 0.191)
Cortisol—mean nmol/L/hour (SD)	0.51 (0.53)	0.61 (0.50)	0.33 (0.49)	*X*^*2*^(2) = 9.52 (*p* = 0.017)

Abbreviations:* KW* Kruskal–Wallis, *HPV* human papilloma virus, *BMI* body mass index, *CRP* c-reactive protein, *IL* interleukin, *TNFα* tumor necrosis factor alpha

Post hoc residuals revealed that the mild class included significantly fewer women (*z* =  − 4.48, *p* < 0.001); fewer oral cavity (*z* =  − 3.01, *p* = 0.039) and more larynx cancer cases (*z* = 4.02, *p* < 0.001); and fewer patients with impaired performance (*z* =  − 2.67, *p* = 0.023), lifetime anxiety (*z* =  − 3.42, *p* = 0.004), and depression (*z* =  − 4.03, *p* < 0.001). On the contrary, the moderate class had significantly more patients with lifetime depression (*z* = 2.71, *p* = 0.020). Finally, the severe class included significantly more women (*z* = 3.74, *p* = 0.001); more patients with impaired performance (*z* = 3.80, *p* < 0.001), lifetime anxiety (*z* = 2.63, *p* = 0.026), and depression (*z* = 2.34, *p* = 0.038); and more daily smokers (*z* = 4.11, *p* < 0.001). As for continuous variables, Dunn’s post hoc tests suggested that patients in the severe class had also higher CRP than those in the mild (*z* = 2.94, *p* = 0.010) and moderate classes (*z* = 2.31, *p* = 0.031) and showed flatter cortisol slopes than the mild (*z* = 2.32, *p* = 0.031) and moderate classes (*z* = 3.09, *p* = 0.006).

### Multinomial logistic regression

Multinomial logistic regression was performed with class regressed on the covariates showing significant differences between classes. When setting the mild class as the reference, patients in both the moderate and the severe classes were more often women (see Table 4). In turn, those in the moderate class had more lifetime history of anxiety and depression, while those in the severe class showed more daily smoking and a flatter cortisol slope, which remained true when compared to the moderate class.

**Table 4:** Odds ratios and 95% confidence intervals for the multivariable logistic regression on the three classes

	Comparison against the mild class OR (95% CI)		Comparison against the moderate class OR (95% CI)
	Moderate	Severe	Severe
Women	3.03 (1.49–6.16)*	3.09 (1.24–7.69)*	1.02 (0.39–2.65)
Oral cavity cancer	1.19 (0.58–2.44)	2.24 (0.89–5.66)	1.88 (0.69–5.09)
Larynx cancer	0.74 (0.35–1.56)	0.40 (0.12–1.36)	0.54 (0.14–2.04)
Performance	1.79 (0.94–3.40)	1.68 (0.71–4.01)	0.94 (0.37–2.39)
Lifetime anxiety	4.33 (1.28–14.67)*	3.17 (0.68–14.69)	0.73 (0.18–3.04)
Lifetime depression	2.57 (1.14–5.81)*	1.71 (0.54–5.38)	0.67 (0.21–2.12)
Daily smoking	0.55 (0.23–1.30)	3.96 (1.60–9.77)*	7.22 (2.47–21.10)*
CRP	1.00 (0.97–1.03)	0.99 (0.95–1.03)	0.99 (0.94–1.03)
Cortisol slope	1.06 (0.61–1.85)	0.41 (0.18–0.93)*	0.38 (0.16–0.93)*

Note: Following between-class comparisons tumor site was collapsed into three categories: oral cavity, larynx, and others

Abbreviations:*OR* odds ratio, *CI* confidence interval, *CRP* c-reactive protein

^*^p < 0.05

## Discussion

Our results underlined that the experience of PNS can be categorized into three severity levels. Up to 40% of our sample suffered from moderate to severe PNS, but a more nuanced look reveals that oral pain and sleep problems were present in all three classes and may therefore be considered symptoms intrinsically related to HNC [40]. In turn, both the moderate and the severe classes stood out for their depression levels, while the moderate was especially affected by anxiety and the severe by fatigue.

Therefore, this study supports the high prevalence of PNS among HNC patients [1, 2]. Indeed, if we focus on previous LCA studies, Tometich et al. [8] featured a two-class model with only 16% of breast cancer patients after diagnosis assigned to the class affected by PNS (mainly represented by anxiety, depression, and fatigue). After treatment, Lewson et al. [15] included one class with no symptoms (52% of the patients), one class with mild anxiety and moderate fatigue (34%), and a class with mild to moderate levels in all PNS except pain (14%) for patients with diverse cancer diagnoses. Han and colleagues [16] distributed colorectal cancer patients in one class with low severity in all PNS (28%); one class with high depression and anxiety (25%); and another with high fatigue, sleep disturbance, and pain (47%). Finally, the three classes retrieved by Harris et al. [17] again in a sample of breast cancer women were labeled as lower (72%), moderate (24%), and higher (4%) PNS.

Our results align with these findings in retrieving three profiles ordered according to PNS severity, with most participants allocated to a class with either mild or no symptoms, and only a minority assigned to the most severe. The only exception is the study by Han et al. [16]. However, this could be explained by the addition of cognitive impairment as an inclusion criterion and later as an indicator variable in the LCA. As for specific symptoms, in these studies, pain and sleep problems were generally less frequent, which reinforces their specific role in HNC. On the contrary, the clear prominence of anxiety, depression, and fatigue supports their general usefulness for mental health screening in cancer.

Regarding our previous network analysis on PNS in HNC [14], poor sleep quality, fatigue, and (to a lesser extent) depression were the core symptoms. Our current study shows that these three symptoms are not experienced equally by all patients, with sleep problems being present in all classes, depression in the moderate and severe, and fatigue only in the severe. This finding may suggest a progressive activation of the network, with more severity associated with sleep problems development into other PNS.

Concerning the covariates analyzed, we found that being a woman, having oral cavity cancer (rather than larynx), worse performance in daily activities, history of depression or anxiety, daily smoking, higher CRP, and flatter cortisol slope were associated with more PNS. Extensive research has shown that women experience more PNS such as anxiety and depression after a cancer diagnosis [41], also for HNC specifically [13, 42]. In turn, pain is the main complaint among oral cavity cancers [40]. Thus, considering the psychosocial implications of this specific diagnosis is important, although the variance explained by tumor site might be shared with other covariates as it lost significance in the multivariable logistic regression. The same applies to worse performance, already associated with higher PNS in previous LCAs [8, 17], but for which mixed results are found in HNC literature [40, 42]. Such inconclusive findings can be reasonably argued to depend on multiple interactions, like tumor site itself [8], which invites to frame this variable in the context of each person.

In the regression, lifetime history of anxiety and depression increased the probability of being assigned to the moderate class, with no differences compared to the severe. This result can be interpreted as such previous experience acting as a proxy to react with anxiety and depression again after the cancer diagnosis [42]. The relevance of both variables suggests that considering previous mental health episodes in screening may be more efficient than asking about specific disorders. As for smoking, it is a widely known risk factor for developing HNC but also for mental health burden after diagnosis [41, 42]. It may be hypothesized that patients who were daily smokers engage in self-blame, leading to higher PNS [43]. However, attention should be given to smoking-related metabolic changes as well, since both daily smoking and flatter cortisol slope were attributable to the severe PNS class. This is aligned with previous literature reporting that smokers experience dysregulations in their HPA activity [44], this reflecting in cortisol alterations, an inflammatory response, and ultimately the experience of depression and other PNS [6, 14, 45]. Indeed, this pathway would also account for the CRP differences between classes.

It is important to highlight that these two biological markers, cortisol and CRP, do not only show a discriminant value between classes here but they were also included in the same cluster as PNS in our previous network analysis [14], underlining their relationship. In fact, they stand out despite the general inflammation observed in the sample: all classes showed mean CRP values > 5 mg/L, when the cut-off flagging inflammation is > 3 mg/L [46]. Therefore, both are proposed as useful PNS biomarkers in cancer. Since systemic inflammation is believed to have prognostic value in HNC [47], and considering that it has also been related to the pathogenesis of a wide range of cancers [48], the interaction between cancer, PNS, and inflammation requires further research.

### Limitations

This study has several limitations. Even if listwise deletion is considered appropriate for MCAR data, our sample may be biased due to the differences between patients included and excluded from analysis. Such differences suggest that our participants have less complexity compared to the original NET-QUBIC sample. Therefore, results need to be interpreted cautiously. Indeed, although this was a multicenter project, it was conducted in a single country, recruited participants diagnosed with a specific group of tumors, and some variables (e.g., ethnicity) were not considered. All these constitute limitations to the generalizability of our results. Additionally, the cross-sectional nature of the study provides an initial description of profiles. However, future longitudinal studies are necessary to assess their evolution over time. Building upon evidence in breast cancer [49], they can be hypothesized to remain stable or even worse during treatment, particularly the severe profile, but such anticipation needs to be tested specifically for HNC.

## Conclusions

Newly diagnosed HNC patients can be classified according to severity of PNS, with 40% suffering from moderate to severe PNS. Oral pain and sleep problems are to be expected in a wide range of patients, with depression and anxiety flagging more complexity, and fatigue being present only in the most severe cases. Special attention should be given to women, patients with oral cavity cancer, worse performance, previous mental health problems, daily smokers, and those with higher CRP and flatter cortisol slope. These factors may serve as drivers for screening routines and efficient care provision, since early detection and proper monitoring of PNS can stop worsening trajectories and the need for further healthcare resources [41]. Future studies are encouraged to investigate if interventions can be tailored to these profiles to pursue higher effectiveness and cost-effectiveness. For example, it can be tested whether the clinical management of pain and sleep disturbances (present in the mild class already) prevents the experience of other PNS, or if patients with an immunometabolic profile, suggested in the severe class, are more responsive to some interventions than others. These results may open the door for more personalized psychosocial treatments, which are needed in the coming years [50].

## Data Availability

The data analyzed in this study is available upon request registration in the NET-QUBIC Data Warehouse and Biobank (https://data.onderzoek.io/kubus/).
